# Diagnostic and treatment delay among pulmonary tuberculosis patients in Ethiopia: a cross sectional study

**DOI:** 10.1186/1471-2334-5-112

**Published:** 2005-12-12

**Authors:** Solomon Yimer, Gunnar Bjune, Getu Alene

**Affiliations:** 1 Amhara National Regional State Health Bureau, Bahir Dar, Ethiopia; 2 Department of General Practice and Community Medicine, University of Oslo, Oslo, Norway; 3 Department of Community Health, University of Gondar, Gondar, Ethiopia

## Abstract

**Background:**

Delayed diagnosis and treatment of tuberculosis (TB) results in severe disease and a higher mortality. It also leads to an increased period of infectivity in the community. The objective of this study was to determine the length of delays, and analyze the factors affecting the delay from onset of symptoms of pulmonary tuberculosis (PTB) until the commencement of treatment.

**Methods:**

In randomly selected TB management units (TBMUs), i.e. government health institutions which have diagnosing and treatment facilities for TB in Amhara Region, we conducted a cross sectional study from September 1-December 31/2003. Delay was analyzed from two perspectives, 1. Period between onset of TB symptoms to first visit to any health provider (health seeking period), and from the first health provider visit to initiation of treatment (health providers' delay), and 2. Period between onset of TB symptoms to first visit to a medical provider (patients' delay), and from this visit to commencement of anti-TB treatment (health systems' delay). Patients were interviewed on the same date of diagnosis using a semi-structured questionnaire. Logistics regression analysis was applied to analyze the risk factors of delays.

**Results:**

A total of 384 new smear positive PTB patients participated in the study. The median total delay was 80 days. The median health-seeking period and health providers' delays were 15 and 61 days, respectively. Conversely, the median patients' and health systems' delays were 30 and 21 days, respectively. Taking medical providers as a reference point, we found that forty eight percent of the subjects delayed for more than one month. Patients' delays were strongly associated with first visit to non-formal health providers and self treatment (P < 0.0001). Prior attendance to a health post/clinic was associated with increased health systems' delay (p < 0.0001).

**Conclusion:**

Delay in the diagnosis and treatment of PTB is unacceptably high in Amhara region. Health providers' and health systems' delays represent the major portion of the total delay. Accessing a simple and rapid diagnostic test for TB at the lowest level of health care facility and encouraging a dialogue among all health providers are imperative interventions.

## Background

Globally, the burden of TB is escalating. Various reasons including poverty, population growth, migration and HIV/AIDS are the major factors for the continued threat of TB in the world, but a significant problem lies with the fact that many cases remain undiagnosed [1]. This could be due to a number of factors, principally found within the categories: patients delaying seeking healthcare or failure of the health care systems to diagnose patients in a timely manner.

Delayed diagnosis may result in more extensive disease [2], more complications and lead to a higher mortality. It also leads to an increased period of infectivity in the community [3]. Delays in the diagnosis of TB have been studied in both high and low income countries and vary significantly from 8.1 weeks in New York [4] to 12 weeks in Botswana [5] and 26 weeks in Tanzania [6]. Individual's perception of the disease, the severity of the disease, access to health services, and the expertise of the health personnel are among factors identified as influencing delay in diagnosis [7].

In Ethiopia, where TB remains to be a major public health threat with increasing incidence of new infectious cases [8], two studies were conducted on diagnostic delay [9,10]. These studies assessed the diagnosis and treatment initiation practice of TB patients in diagnosing facilities. The roles of other providers in delaying a TB patient have not been studied in Ethiopia. The objective of the current study was to investigate diagnostic and treatment delays from two perspectives. All health providers and only medical providers have been taken separately as reference points to determine the length and associated risk factors of patients', health systems' and health providers' delay among new smear positive PTB patients in Amhara region, Northwest Ethiopia.

## Methods

An institution based cross-sectional study was conducted between September 1, 2003 and December 31, 2003 among new smear positive PTB patients in Amhara region, the second largest region of the country with a population of 18.2 million [11].

To select representative sample for the study, first we selected 6 zones randomly out of the 11 zones of the study region followed by listing all TBMUs in the selected zones, then we took 20 TBMUs randomly as a study sites. Finally, we interviewed the study subjects right after diagnosis consecutively until the intended sample size was achieved. New smear positive PTB patients above 15 years of age were included in the study. Smear negatives, relapsed or failed treatment were excluded.

The sample size was calculated using the formula required for determination of sample size for estimating single proportions [12]. Therefore, by taking a previous study done on diagnostic delay in Ethiopia, which showed 58% proportion of delay of more than one month [9], and a 95% confidence interval and a margin error of 5%, the sample size was calculated to be 373. We studied 384, which hold 103 % of the estimated sample size.

A pre-tested semi-structured questionnaire was administered to collect the intended data. Questions assessed, socio-demographics, major presenting symptoms of PTB, duration of major presenting symptoms and the date of first health care visit for each enrollee. The major pulmonary symptoms asked were presence of cough for more than 3 weeks, production of sputum, chest pain and haemoptysis. Questions regarding knowledge of TB and stigma were also included. Health officers working in the study area interviewed the subjects immediately following the diagnosis. During the interview, if a patient had anorexia for over a year, but was seeking medical care for a cough of one-month duration, the latter was taken as the duration of illness. Patient register cards, TB registration books and laboratory registries were crosschecked to assure the quality of data.

The National TB control programme in Ethiopia predominantly uses passive case finding as a system for detecting PTB cases. The recommended standard procedures applied in the diagnosis of PTB are to collect and examine three sputum specimens from individual patients with respiratory symptoms in two consecutive days [8]. Examination of sputum by direct microscopy for the presence of acid fast bacilli (AFB) is performed at health facilities designated as diagnostic and treatment centers of all self presenting persons with symptoms suggestive of TB. Pulmonary positive is confirmed when there are at least 2 AFB positive smear results or when one sputum specimen is positive for AFB in addition to radiographic abnormalities consistent with active PTB [8].

### Definition of variables

**Figure 1:**
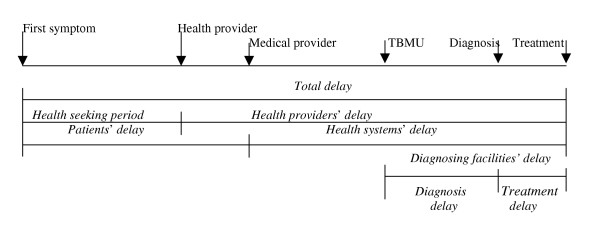
**The relation of the different delay periods**. This figure indicates the relations of the different delay periods and the possible health providers visit, be it formal or non formal. It also indicates the operational definitions for the delay periods. For example, the second line from above shows the definition for the total delay, which is the delay period from the onset of cough until the commencement of anti- TB treatment.

*Health provider *is defined as any individual consulted by the patient about his / her illness that gave or prescribed something (whatever the form) for treatment (excluding the family)[7]. *Formal (medical) health providers are *health centers, hospitals & clinics owned by the government or the private sectors. *Non-formal health providers are *traditional health care providers, drug retail outlets and local injectors. *Drug retail outlets are *pharmacies, drug stores, drug venders and open market drug sellers. The relations of patients', health providers', health systems', diagnosing facilities' and total delays are shown in figure 1.

### Statistical analysis

SPSS version 11.0 was used for analysis. We took 30 days for patients' and 15 days for health systems' delay as cut off points to dichotomize the sample in to either shorter or longer delay period. Thirty days was chosen as it was the median for patients' delay. However, the decision to use 15 days was based on consultation made with treating physicians and using the experience of the previous Ethiopian study on the same area [10].

As the data were skewed, group differences were compared using the non-parametric Mann-Whitney and Kruskal-Wallis tests. Responses to questions that assessed TB knowledge and stigma related to HIV/TB were analyzed by calculating their means and interquartile scores, and finally, the responses were categorized into high/low knowledge or high/low stigma and were cross-tabulated with the main outcome variables for possible associations. Univariate and multivariate (logistic) regression analysis were performed to assess the relative impact of predictor variables on the outcome variables, and a p-value of < 0.05 was considered statistically significant.

## Results

### Patient characteristics

A total of 384 new smear positive PTB patients were enrolled in a four months study period, 52.6% males and 47.4% females. The mean age was 29.8 years, range being 16–70 years with a median age of 28 years. Among the study subjects 56.3% lived beyond 10 km from a medical facility. Occupationally, 27.1% were farmers, 22.4% housewives, 15.4% civil servants, 14.3% unemployed, 11.7 % self-employed and 9.1 % were students. Income distribution among the study population showed that 44.3% did not have defined income, 27.1% had irregular income, 15.3% had regular income of US$0.1–35 per month and 13.3% had income of more than US$36 per month. With regards to education, 40.9% of the study population were illiterate, 37.8% had completed 1–8^th ^grade and 2.6% had completed 12 grade and above. Approximately 34.9% of the respondents were never married, while 36.5 % were married, 23% divorced and 6% widowed.

### Symptoms

The major symptoms that patients experienced during the onset of their illness included, cough (96.4%), followed by tiredness (92.2%), weight loss (90.2%), loss of appetite (86.5%), night sweating (85.2%) chest pain (79.4%) fever (76%) and haemoptysis (25%).

### Delay, taking all health providers as a reference point

#### Health seeking period

**Figure 2:**
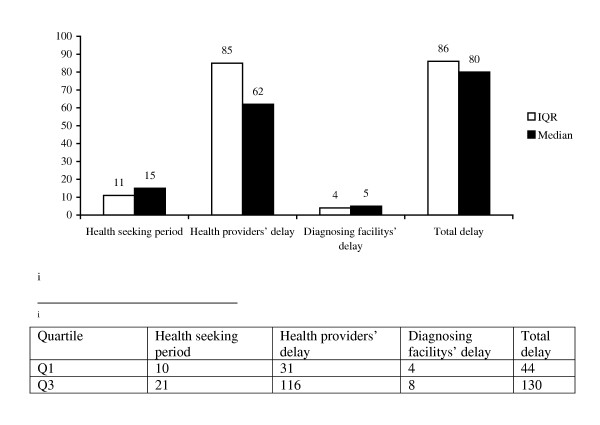
**Median delay period and inter quartile range (IQR) taking all health providers as a reference point**. This figure shows the median delay periods taking all health providers as a reference points. It divides the delay periods in to four, namely health seeking period, health providers' delay, diagnosing facilities' delay and total delay. As can be seen from the figure, patients took 15 days to first visit a health provider after the onset of their cough. This indicates that patients seek for treatment relatively early irrespective of the type health provider visited. On the other hand the median health providers' delay was very long which shows the possible area for intervention activities.

Considering all health providers as a reference point, the median health-seeking period was 15 days IQR (15–21 days) (Figure 2). Almost all of the patients had visited a health care provider within one-month time from the onset of their symptoms. Of all the respondents, 61.7% initially visited non-formal health providers (including traditional healers 27.1%, drug retail outlets 31% and local injectors 3.6%). On the other hand, 38.3% visited the formal medical providers (including government health posts/clinics 9.4%, health centers 15%, government hospitals 3.9%, and private clinics 9.9%). The decision about where to go for help was most influenced by close family members (89%). Friends and health professionals also influenced patients' decision to seek care (11%).

#### Health providers' delay

The median health providers' delay was 61 days (IQR 31–116 days) (Figure 2). Health providers' delay did not vary by sex, marital status or income of the patients. In logistic regression analysis, those who lived within 10 Km radius of a medical facility (OR = 0.42 95%CI, 0.24, 0.72), went to school 1–8^th ^grade (OR = 0.56 95%CI, 0.33, 0.97), were 9^th ^grade and above (OR = 0.40 95%CI, 0.20, 0.81) and those who attended formal health providers initially (OR = 0.35 95%CI, 0.20, 0.81) were less likely to have longer health providers' delay (See additional file 1).

### Delay, taking medical providers as a reference point

#### Patients' delay

**Figure 3:**
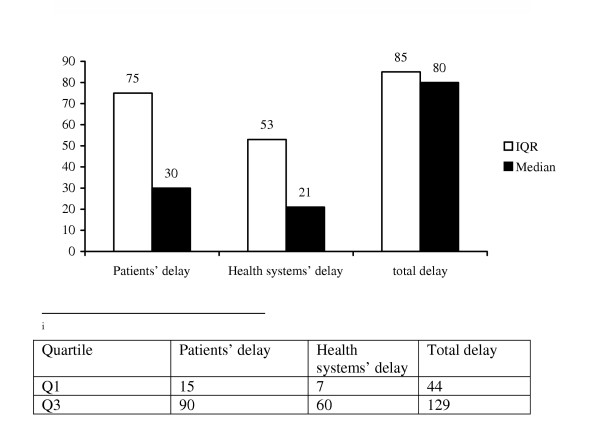
**Median delay periods and inter quartile range (IQR) taking only medical providers as a reference point**. This figure tries to show the median delay period taking only medical providers as a reference point. The delay periods are divided in to three, namely patients', health systems' and total delay periods. In this figure, median patients' delay is higher compared to the median health-seeking period in figure 2, which shows that patients take relatively longer time until they consult medical providers that play a crucial role for early diagnosis of TB.

Considering only medical providers as a reference point, the median delay from onset of cough to first visit to a medical provider was 30 days, IQR (15–90 days) (Figure 3). Based on the cumulative distribution 52% of the subjects consulted a medical provider within 30 days of the onset of their illness, whereas for 48% of the respondents it took more than 31 days. The longest delay was reported to be 2 years.

The median patients' delay varied with the patients' area of residence. Those who lived within 10 Km radius of a medical facility reported earlier compared to those living beyond 10 Km (Mann-Whitney test; P < 0.001). Patients that initially visited non-formal health care providers and those who self-treated themselves had longer median patients' delay compared to those who went directly to the first medical provider (Mann-Whitney test; P < 0.001).

In multivariate logistic regression analysis (See additional file 2), those who lived beyond 10 Km radius of a medical facility (OR = 3.81, 95%CI 2.21–6.57), age>45 years (OR = 2.62, 95% CI 1.13–6.02) and self-treatment (OR = 1.69, 95% CI 1.04–2.75) were significantly associated with increased patients' delay.

#### Health systems' delay

The median health systems' delay was 21 days, IQR 7–60 days (Figure 3). There was no difference according to socio-demographic factors. However, significant differences were observed with type of first medical provider visited. Those first visiting a health post/clinic or a private medical provider experienced longer health systems' delay compared to those who visited government health centers or hospitals (Kruskal-Wallis test; P < 0.001).

Logistics regression analysis showed that those who first visited a health post/clinic (OR = 3.50, 95%CI 1.86–6.57) or a private medical provider (OR = 2.10 95%CI 1.18, 3.71) were significantly associated with increased health systems' delay (See additional file 3).

#### Diagnosing facilities' delay

Several providers referred patients to the TBMUs. In general, government health assistants, nurses and doctors referred 271 (70.6%) of the patients. Private medical providers referred 92 (24.0%), 11 (2.9%) were self-referred, 8 (2.1%) were referred by friends, and 2 (0.5%) were referred by pharmacists. Overall, the median diagnosing facilities' delay was 5 days, IQR (4–8 days). This includes a median diagnosis delay of 3 days and treatment delay of 2 days. The longest diagnosing facilities' delay was reported to be 31 days.

#### Total delay

The median total delay was 80 days IQR (44.2- 129.8 days). The cumulative distribution showed that only 9% of the total respondents were detected and put on treatment within one month of the onset of their illness where as for 91% of the respondents the total delay exceeded 31 days.

## Discussion

In this study by aggregating all health providers and only medical providers into two separate reference points, we were able to observe variations in the length of the different delay periods. Patients who first visited a *medical provider *were delayed for 21 days before they initiated treatment. On the other hand, cases who first visited a *health provider *had taken 15 days to first seek health care, whereas the period from first visit to a health provider to first initiation of treatment was 4 folds of the health-seeking period. This clearly suggests a potential area for possible interventions.

Taking all health providers as a reference point, the median health-seeking period and health providers' delay observed in this study is quite similar with the study conducted in the Gambia [7], Alternatively, considering only medical providers as a reference point, the health systems' delay showed a median delay of 21 days, which is more or less similar with other studies conducted in Tanzania [6], Penang [13], New York [14] and Japan [15] that showed a median delay of 3 weeks to 1 month.

Prior attendance to a health post or clinic was a risk factor for longer health systems' delay. Similar findings were done in Botswana [5]. Our findings can be explained by the fact that, health posts and clinics are not equipped with diagnostic facilities for TB. In addition, the facilities are run by health assistants and junior nurses whose primary training is not to diagnose serious diseases but to concentrate on patient care and preventive activities. First visit to private medical providers was also identified as a risk factor for longer health systems' delay. In this regard, a similar finding was observed in Penang [13]. Other studies have documented an increased health systems' delay among females compared to males [3,16]. This finding could not be confirmed in our study.

In the present study, the median patients' delay was 30 days. This is in accordance with other studies that were conducted in Botswana [5], Ghana [3], Philippines [17] and Penang [13], which showed a median patients' delay of 3 – 4 weeks. Patients' delay was significantly associated with older age (>45 years) and distance (>10 km) from a health care facility. These have proved to be important factors in other studies in Zambia [18] and south Ethiopia [9]. Our findings might be related to the fact that older people may rely more heavily on other persons, which makes it difficult for them to visit health facilities at preferred time. We observed a significant association between self-treatment and longer patients' delay. This is similar with studies conducted in other African countries such as Ghana [3], Botswana [5] and Kenya [19], which might be related to poor knowledge of TB among the population.

The median diagnosing facilities' delay observed in this study was 5 days. This is somewhat higher than the Gambian study that showed a median delay of 2 days [7]. The reason could be related to the low health service coverage (50%) in Ethiopia [8] unlike in Gambia where 87% of the population has a good access to health care [7]. However, we believe that the diagnosing facilities' delay observed in our study is not that wide. Therefore, we may say that patients get their diagnosis and commence their treatment within a reasonable period of time as long as they manage to reach the TBMU.

The current study has potential limitations. First we studied patients from government health care facilities. Similar patients who might have gone to other health providers and stayed at home during the study period were not included, thus making our result difficult to generalize to all smear positive PTB patients in the region. Second, our outcome measure of delay in seeking care was self-reported, implying a recall bias. To minimize this problem, we specifically asked the onset of the major symptoms and how long after these symptoms they consulted a health provider. Moreover we have used local calendar listing the main religious and national days to estimate the date of onset of symptoms.

## Conclusion

In general, our study showed a significant delay in the initiation of treatment among smear positive PTB cases in Amhara region, Northwest Ethiopia. To our surprise we found that patients seek health care relatively early. The major factors associated with the patients' delay were related to lower access to medical providers and prior attendance to non-formal health providers. In contrast, the major factors associated with the health systems' delay were prior attendance to the health posts/clinic and private medical providers. Therefore, considering the high magnitude of pretreatment delay, it is imperative to access a simple and rapid diagnostic test for TB that can be used at the lowest health care facility level in the region. This might shorten the long health systems' delay. Besides this, encouraging a dialogue among the non formal health providers and private medical providers, improving access to DOTS at the periphery and educating the public to raise the awareness on the symptoms and treatment of TB should be given due emphasis.

## Competing interests

The author(s) declare that they have no competing interests.

## Authors' contributions

**Author 1**. SA: Initiated the research, wrote the research proposal, conducted the research, did data entry and analysis and wrote the manuscript.

**Author 2**. GB: Served as main supervisor and was involved in the write up of the proposal, in the data analysis and write up of the manuscript

**Author 3**. GA: Served as co-supervisor and was involved in the data analysis and write up of the manuscript

## Pre-publication history

The pre-publication history for this paper can be accessed here:



## Supplementary Material

Additional File 1 **Associations of soci-demographic and health service factors with health providers' delay**. In this table the associations of soci-demographic and health service factors with health providers' delay have been analyzed. It shows that patients who lived within 10 Km radius of a medical facility, those who were 9^th ^grade and above and those who attended formal health providers initially were less likely to have longer health providers' delay. Click here for file

Additional File 2 **Associations of socio-demographic and health service factors with patients' delay**. From this table we can see that those who lived beyond 10 Km radius of a medical facility, those who were >45 years age and who used self-treatment had increased risk of patients' delay. Click here for file

Additional File 3 **Associations of socio-demographic and health service factors with health systems' delay**. This table shows the association of socio-demographic and health services factors with health systems' delay. We found that patients who first visited a health post/clinic or a private medical provider were significantly associated with increased health systems' delay. Click here for file
