# Supplementary material for: Diagnostic and treatment delay among pulmonary tuberculosis patients in Ethiopia: a cross sectional study
Source: BMC Infect Dis. 2005 Dec 12;5:112. doi: 10.1186/1471-2334-5-112 (PMC1326202; doi:10.1186/1471-2334-5-112)
Supplement: Additional File 1 — Associations of soci-demographic and health service factors with health providers' delay. In this table the associations of soci-demographic and health service factors with health providers' delay have been analyzed. It shows that patients who lived within 10 Km radius of a medical facility, those who were 9th grade and above and those who attended formal health providers initially were less likely to have longer health providers' delay. [file 1471-2334-5-112-S1.pdf]

Table 1 Associations of soci-demographic and health service factors with health providers' delay

Characteristics	Delay >62 days	No delay ≤61 days	Crude OR (95%CI)	Adjusted OR (95%CI)
**Sex**				
Male	94	108	1.00	1.00
Female	96	86	1.39 (0.93, 2.08)	0.89 (0.51, 1.53)
**Age**				
15-24	47	80	1.00	1.00
25-44	119	97	0.20 (1.33, 3.27) *	2.04 (1.21, 3.4) *
>45	24	17	2.40 (1.17, 4.92) *	2.02 (0.88, 4.60)
**Residence**				
>10Km	110	58	1.00	1.00
≤10Km	80	136	0.31 (0.21, 0.47) *	0.42 (0.24, 0.72) *
**Occupation**				
Farmers	60	45	1.00	1.00
Housewives	55	42	1.31(0.72, 2, 34)	0.87 (0.32, 2.44)
Civil servants	21	38	0.41(0.21, 0.78) *	0.45 (0.13, 1.53)
Students	9	24	0.25 (0.11, 0.59) *	0.33 (0.10, 1.08)
Unemployed	27	23	0.71(0.37, 1.36)	0.78 (0.29, 2.09)
Self employed	18	27	0.48 (0.24, 0.99) *	0.45 (0.15, 1.3)
**Marriage**				
Single	59	75	1.00	1.00
Divorced	39	50	0.99 (0.57, 1.70)	0.89 (0.45, 1.6)
Married	85	55	1.96 (1.2, 3.17)	1.61 (0.87, 2.94)
Widowed	7	14	0.64 (0.24, 1.67)	0.60 (0.19, 1.83)
**Education**				
Illiterate	102	55	1.00	1.00
1-8th grade	65	80	0.43 (0.27, 0.69) *	0.56 (0.33, 0.97) *
9th and above	23	59	0.21 (0.12, 0.37) *	0.40 (0.20, 0.81) *
**Health provider visit**				
Non-formal	143	94	1.00	1.00
Formal	47	100	0.31 (0.20, 0.47) *	0.35 (0.21, 0.57) *
**Income**				
No income	98	87	1.00	1.00
Irregular income	56	58	0.85 (0.54, 1.36)	0.65 (0.28, 1.54)
1-300 Birr	21	15	1.24 (0.60, 2.56)	2.11 (0.77, 5.7
>301Birr	15	34	0.39 (0.20, 0.76) *	0.79 (0.26, 2.41)
**Medical provider**				
Clinic/HP	38	39	1.00	1.00
Health center	86	77	1.14 (0.67, 1.97)	1.08 (0.56, 2.06)
Hospital	25	27	0.95 (0.47, 1.92)	1.29 (0.56, 3.00)
Private medical provider	41	51	0.89 (0.45, 1.51)	1.26 (0.61, 2.60)

* Significant at <0.05

Income group: 1) no income (housewife, students, unemployed); 2) irregular income (farmers); 3) regular income of 1-300 Birr per month (civil servants); 4) regular income of >300 Birr per month (civil servants).
