# Supplementary material for: Diagnostic and treatment delay among pulmonary tuberculosis patients in Ethiopia: a cross sectional study
Source: BMC Infect Dis. 2005 Dec 12;5:112. doi: 10.1186/1471-2334-5-112 (PMC1326202; doi:10.1186/1471-2334-5-112)
Supplement: Additional File 2 — Associations of socio-demographic and health service factors with patients' delay. From this table we can see that those who lived beyond 10 Km radius of a medical facility, those who were >45 years age and who used self-treatment had increased risk of patients' delay. [file 1471-2334-5-112-S2.pdf]

Table 2 Associations of socio-demographic and health service factors with patients' delay

Characteristics	Delay >31 days	No delay ≤30 days	Crude OR (95%CI)	Adjusted OR (95%CI)
**Sex**				
Male	91	111	1.00	1.00
Female	94	88	1.19 (0.80, 1.79)	1.01 (0.62, 1.91)
**Age**				
15-24	49	78	1.00	1:00
25-44	110	106	1.65 (1.08, 2.58) *	1.47 (0.86, 2.49)
>45	26	15	2.76 (1.33, 5.72) *	2.62 (1.13, 6.09) *
**Residence**				
>10Km	112	56	3.92 (2.56, 6.00) *	3.81 (2.21, 6.57) *
≤10Km	73	14	1.00	1:00
**Occupation**				
Farmers	59	45	1.00	1:00
Housewives	44	42	0.79 (0.45, 1.42)	1.36 (0.12, 1.03)
Civil servants	21	38	0.42 (0.22, 0.82) *	0.61 (0, 17.2.19)
Students	11	24	0.35 (0.16, 0.79) *	0.40 (0.12, 1.32)
Unemployed	32	23	1.06 (0.55, 2.06)	0.96 (0.35, 2.66)
Self employed	18	27	0.51 (0.25, 1.03)	0.78 (0.26, 2.34)
**Marriage**				
Single	59	75	1.00	1.00
Divorced	45	44	1.30 (0.76, 2.22)	1.39 (0.72, 2.67)
Married	74	66	1.43 (0.89, 2.29)	1.30 (0.69, 2.42)
Widowed	7	14	0.64 (0.24, 1.68)	0.69 (0.22, 2.16)
**Education**				
Illiterate	91	66	1.00	1.00
1-8th grade	70	75	0.68 (0.43, 1.07)	1.38 (0.67, 2.83)
9th and above	24	58	0.30 (0.17, 0.53) *	1.47 (0.75, 2.87)
**Health provider visit**				
Formal	47	100	1.00	1.00
Non-formal	138	99	2.97 (1.93, 4.57) *	0.34 (0.20, 0.57) *
**Income**				
No income	100	85	1.00	1.00
Irregular income	54	60	0.77 (0.48, 1.22)	1.30 (0.43, 3.90)
1-300 Birr	14	22	0.54 (0.26, 1.12)	0.57 (0.16, 1.98)
>301birr	17	32	0.45 (0.23, 1.87)	0.49 (0.17, 1.46)
**Self-treatment**				
Yes	121	59	1.75 (1.16, 2.62) *	1.69 (1.04, 2.75) *
No	110	94	1.00	1.00
**Knowledge of TB**				
Low	100	60	2.72 (1.79, 4.14) *	1.89 (1.15, 3.10) *
High	85	139	1:00	1:00
**Stigma**				
Low	67	66	1:00	1:00
High	118	133	0.87 (0.57, 1.33)	0.88 (0.54, 1.45)

Significant at <0.05

Income group: 1) no income (housewife, students, unemployed); 2) irregular income (farmers); 3) regular income of 1-300 Birr per month (civil servants); 4) regular income of >300 Birr per month (civil servants).
