# Supplementary material for: Diagnostic and treatment delay among pulmonary tuberculosis patients in Ethiopia: a cross sectional study
Source: BMC Infect Dis. 2005 Dec 12;5:112. doi: 10.1186/1471-2334-5-112 (PMC1326202; doi:10.1186/1471-2334-5-112)
Supplement: Additional File 3 — Associations of socio-demographic and health service factors with health systems' delay. This table shows the association of socio-demographic and health services factors with health systems' delay. We found that patients who first visited a health post/clinic or a private medical provider were significantly associated with increased health systems' delay. [file 1471-2334-5-112-S3.pdf]

Table 3 Associations of socio-demographic and health service factors with health systems' delay

Characteristics	Delay >16 days	No delay ≤15 days	Crude OR (95%CI)	Adjusted OR (95%CI)
**Sex**				
Male	117	85	1.00	1.00
Female	103	79	0.95 (0.63, 1.42)	0.73 (0.44, 1.21)
**Age**				
15-24	63	64	1.00	1:00
25-44	133	83	1.65(1.08, 2.58) *	1.62 (0.98, 2.61)
>45	24	17	2.76 (1.33, 5.72*	1.30 (0.59, 2.90)
**Marital status**				
Single	79	55	1:00	1:00
Divorced	52	37	0.97 (0.56, 1.68)	1.12 (0.61, 2.05)
Married	76	64	0.83 (0.51, 1.33)	0.91 (0.51, 1.64)
Widowed	13	8	1.13 (0.94, 2.91)	1.12 (0.38, 3.26)
**Occupatio**n				
Farmers	46	58	1.00	
Housewives	26	60	1.80 (1.00, 3.33)	2.16 (0.84, 5.5)
Civil servants	31	28	0.71 (0.37, 1.36)	0.74 (0.26, 2.17)
Students	17	18	0.84 (0.39, 1.80)	0.68 (0.24, 1.93)
Unemployed	23	32	1.10 (0.57, 2.13)	0.92 (0.37, 2.31)
Self-employed	21	24	0.90 (0.44, 1.82)	0.64 (0.24, 1.70)
**Income**				
No income	73	112	1.00	
Irregular income	50	64	0.83 (0.52, 1.33)	0.91 (0.43, 1.94)
Income1-300birr	14	22	1.0 0(0.49, 2.13)	1.62 (0.61, 4.18)
>301	27	22	0.53 (0.28, 1.00)	0.54 (0.19, 1.48)
**Residence**				
>10Km	90	78	1.00	1.00
≤10Km	130	86	1.31 (0.87, 1.97)	2.01 (1.22, 3.48) *
**Education**				
Illiterate	90	67	1.00	1.00
1-8th grade	85	60	1.06 (0.67, 1.67)	1.00 (0.59, 1.69)
9th and above	45	37	0.90 (053, 1.55)	0.64 (0.33, 1.23)
**Medical provider**				
Health center	79	84	1.00	1.00
Health post	57	20	3.03 (1.67, 5.49) *	3.90 (2.06, 7.64) *
Hospital	23	29	0.84 (0.45, 1.58)	0.82 (0.41, 1.62)
Private	61	31	2.32 (1.23, 3.55) *	2.32 (1.23, 4.23) *

* Significant at< 0.05

Income group: 1) no income (housewife, students, unemployed); 2) irregular income (farmers); 3) regular income of 1-300 Birr per month (civil servants); 4) regular income of >300 Birr per month (civil servants).
